# Evaluation of Cardiac Parameters in Bone Marrow Transplant Patients: Effect of Pulmonary Artery Pressure on Survival

**DOI:** 10.4274/tjh.galenos.2018.2018.0015

**Published:** 2019-02-07

**Authors:** Ali Caner Özdöver, İlknur Gündeş, Melya Pelin Kırık, Handan Haydaroğlu Şahin, Murat Sucu, Mustafa Pehlivan

**Affiliations:** 1 Gaziantep University Faculty of Medicine, Department of Internal Medicine, Gaziantep, Turkey; 2 Gaziantep University Faculty of Medicine, Department of Hematology, Gaziantep, Turkey; 3 Gaziantep University Faculty of Medicine, Department of Cardiology, Gaziantep, Turkey; 4 Gaziantep University Faculty of Medicine, Department of Hematology, Bone Marrow Transplant Unit, Gaziantep, Turkey

**Keywords:** Hematopoietic stem cell transplantation, Pulmonary artery pressure, Overall survival

## Abstract

**Objective:**

Hematopoietic stem cell transplantation (HSCT) is a choice of treatment for malignant and non-malignant diseases. After HSCT, some complications may develop in patients. Cardiac complications are particularly important. The aim of this study was to investigate whether systolic pulmonary artery pressure (PAP) is a marker for overall survival (OS) in HSCT patients.

**Materials and Methods:**

In our study, 428 HSCT patients were evaluated. Ejection fraction (EF) and PAP values were investigated during symptom-oriented echocardiography in the pre-HSCT and post-HSCT periods.

**Results:**

Pre-HSCT EF values were similar between the groups. In patients with autologous HSCT (auto-HSCT) (PAP >25 mmHg), it was found that the 5-year mortality rate was 48.6%, while in the other group (PAP <25 mmHg) the 5-year mortality was 25.5%. There was a significant association between 5-year mortality rate and PAP level (p<0.046) in the auto-HSCT group. OS was 38% in the pre-auto-HSCT period with PAP values of >25 mmHg, while OS was 61% in the pre-auto-HSCT period with PAP values of <25 mmHg (p<0.001). We determined that there was a statistically significant difference between OS and PAP levels in patients with auto-HSCT. Five-year mortality rate and OS were not significantly different in patients undergoing allogeneic HSCT (allo-HSCT) (p>0.05).

**Conclusion:**

Our results suggest that pre-HSCT PAP value is an important risk factor for mortality and OS in patients undergoing auto-HSCT.

## Abstract (tr)

**Amaç:**

Hematopoietik kök hücre transplantasyonu (HKHT) malign ve non-malign hastalıklarda kullanılan bir tedavi seçeneğidir. Nakil sonrasında bir çok komplikasyon gelişmektedir. Kardiyak komplikasyonlar önemli bir yer tutmaktadır. Bu çalışmanın amacı, sistolik pulmoner arter basıncının (PAB) nakil yapılan hastaların sağkalımına olan etkisini araştırmaktır.

**Gereç ve Yöntemler:**

Çalışmamızda 428 HKHT yapılan hasta değerlendirildi. Ejeksiyon fraksiyonu (EF) ve PAB değerleri, semptom oryante ekokardiyografi ile post ve pretransplantasyon dönemde değerlendirildi.

**Bulgular:**

Karşılaştırılan gruplar arasında pre-HKHT döneminde EF değerleri açısından farklılık saptanmadı. Beş yıllık mortalite otolog HKHT yapılan hastalarda PAB değeri 25 mmHg üzeri olanlarda %48,6, diğer grupta %25,5 olarak gözlendi ve istatistiksel olarak p<0,046 olarak hesaplandı. Total sağkalıma bakıldığında otolog HKHT yapılan hastalarda; nakil öncesi PAB değeri 25 mmHg üzeri olanlarda %38 iken; nakil öncesi PAB değeri 25 mmHg altında olan grupta sağkalım %61 olarak saptandı ve bu durum istatistiksel olarak anlamlı hesaplandı (p<0,001). Aynı verileri değerlendirerek total sağkalım ve PAB değeri arasında otology HKHT yapılan hastalarda anlamlı ilişki oldugunu, allojenik HKHT yapılanlar arasında istatistiksel olarak anlamlı ilişki olmadığını tespit etmiş bulunmaktayız.

**Sonuç:**

Çalışmamızın sonuçlarına göre HKHT öncesi ve sonrası ölçülen PAB değeri hastalarda total sağkalım ve mortalite üzerine önemli etkisi olan bir faktör olarak karşımıza çıkmaktadır.

## Introduction

Hematopoietic stem cell transplantation (HSCT) is used in the treatment of life-threatening malignant and non-malignant diseases. Allogeneic HSCT (allo-HSCT) can provide an advantage for overall survival (OS) in 15%-20% of patients with acute leukemia after induction therapy and this rate may increase to 35% when HSCT is applied during the first relapse and second remission [1]. Autologous HSCT (auto-HSCT) is a good choice of treatment for multiple myeloma; it can be applied in first- and second-line treatment and can also be used in patients with lymphoma as an effective treatment [1].

Short- and long-term complications can develop after HSCT. These include nausea, vomiting, pneumonia, thyroiditis, and cardiovascular side effects [2]. Cardiac complications such as pericarditis, arrhythmia, pulmonary edema, heart failure, and sudden cardiac death developing within the first 100 days of HSCT are considered as acute cardiotoxicity. Studies have shown that post-transplant acute cardiac complications have 1.2% mortality and morbidity ranging from 5% to 43% [3].

Heart failure (HF) is the most serious of cardiac complications. HF is defined as a 10% decrease in the ejection fraction (EF) or EF of less than 50% before HSCT [4]. Clinical findings such as orthopnea, paroxysmal nocturnal dyspnea, exercise intolerance, night cough, wheezing, palpitations, and syncope may develop in HF. To date, there is no medical treatment that can regenerate scar tissue in the routine management of HF, thus increasing mortality and morbidity [5]. It is therefore important to detect HF early. In our study, all of the patients were evaluated for HF with echocardiography. 

Pulmonary hypertension (PH) is defined as pulmonary artery pressure (PAP) of more than 25 mmHg at rest. Right heart catheterization is used as the gold standard in diagnosis, but this method is not suitable for daily practical use [6]. In the past 30 years, prodigious technological improvements in echocardiography have increased its sensitivity for quantifying PAP and it is now used as a safe and available alternative to invasive catheterization [7].

To date, elevation of PAP has not been reported among the cardiac complications in the European Group for Blood and Marrow Transplantation (EBMT) guidelines. In the literature, the focus is generally on PH and mortality in the post-transplant period. A study by Dandoy et al. [8] revealed that symptoms of newly developed tachypnea, hypoxia, and respiratory failure occurred following transplant. It was reported that the mortality rate in 40 cases presented in the literature was 55% and the cause of mortality was PH and its complications in 86% of those cases.

The aim of this study was to investigate whether PAP is a marker of OS for HSCT patients.

## Materials and Methods

### Research Strategy

In this study, 428 patients who underwent HSCT in the Bone Marrow Transplantation Unit of the Department of Hematology, Faculty of Medicine, at Gaziantep University in Turkey were investigated. The median age was 29 (range: 15-63) years in allo-HSCT patients and 54 (range: 17-76) years in the auto- HSCT group. Males comprised 250 (58.4%) of the patients and 178 (41.6%) were female. In this retrospective analysis, the patients who underwent HSCT once between 2009 and 2016 were evaluated using the official Medulla system and by scanning files from the archives. Ethics committee approval was obtained from the Gaziantep University Medical Faculty Ethics Committee with decision number 4.4.2016/103. This study was conducted in accordance with the World Medical Association’s 2000 Declaration of Helsinki.

### Methods

EF and PAP values were evaluated during symptom-oriented echocardiography (Vivid 9, GE, Norway) examinations performed in the pre-HSCT and post-HSCT periods. Continuous wave Doppler imaging of the tricuspid regurgitation (TR) trace was used to measure the difference in pressures between the right ventricle and right atrium. The simplified Bernoulli equation (p=4[TRmax]2) was used to calculate this pressure difference using peak TR velocity. This method correlates well with systolic PAP for right heart catheterization [9]. The cut-off EF value was 50% and that of PAP was 25 mmHg according to the 2015 European Society of Cardiology/European Respiratory Society Guidelines for the diagnosis and treatment of PAP [10].

### Statistical Analysis

The analysis of data was performed using SPSS 18.0 for Windows (SPSS Inc., Chicago, IL, USA). The statistical significance of the differences between the patient groups was estimated by logistic regression analysis. The adjusted odds ratios (ORs) were calculated with a logistic regression model that checked for sex and age and are reported with 95% confidence intervals (CIs). 

Differences in the patients group were compared using the chi-square test and the Fisher exact test when required. For statistical comparison of groups, the median test was used. OS was defined as the time period between the time of transplantation to death due to any reason. OS evaluations were performed using the Kaplan-Meier method. A p-value of less than 0.05 was accepted as significant.

## Results

Allo-HSCT was performed for 154 (36%) and auto-HSCT was performed for 274 (64%) of the 428 patients who were enrolled in this study. General characteristics of the patients are summarized in Table 1. The patients who had undergone auto-HSCT were mostly diagnosed with multiple myeloma 175 (40.9%), followed by 87 (22.4%) with non-Hodgkin lymphoma. A total of 92 (21.5%) patients (80 allo-HSCT and 12 auto-HSCT cases) were diagnosed with acute myeloid leukemia, being the third most common disease group. The final group of patients consisted of 27 (6.3%) patients with aplastic anemia (Table 1). When the follow-up period was taken into account, it lasted between 3 and 84 months (median: 24 months) in the allo-HSCT group and between 3 and 88 months (median: 30 months) in the auto-HSCT group.

In patients who underwent allo-HSCT, pre-transplant EF values were between 43% and 67% (median: 60%), while EF values in patients with auto-HSCT ranged between 35% and 70% (median: 60%). In the post-transplant period, 105 patients were evaluated using echocardiography, and 3 (2%) patients in the allo-HSCT group and 8 patients (2.9%) in the auto-HSCT group had lower EF values before transplantation. As a result of this study, it was found that EF values increased by more than 10% in post-transplant evaluations in 2 out of 45 patients (4.4%) in the allo-HSCT group and 2 out of 60 patients (3.3%) in the auto-HSCT group. In stem cell treatment, multipotent bone marrow-derived mesenchymal stem cells appear to be the most promising candidates. Bone marrow-derived mesenchymal stem cells increase proliferation and attenuate scar tissue [11]. The EF increases detected in our study were thought to be due to regeneration.

**Figure 1:**
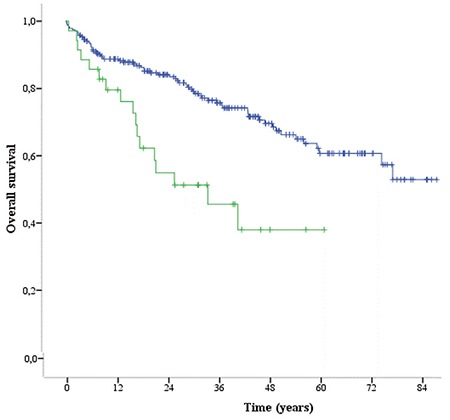
Five-year overall survival (38% vs. 61%) for the whole population according to pulmonary artery pressure in autologous hematopoietic stem cell transplantation group.

PAP was higher than 25 mmHg in 35 patients (12.8%) in the auto-HSCT group and in 22 (14.3%) in the allo-HSCT group. In the pre-transplant period, 105 patients were evaluated using percentage of forced expiratory volume in one second (FEV1 %), with a median of 83% (80%-95%) in the allo-HSCT group and 89% (82%-97%) in the auto-HSCT group. Tricuspid and mitral regurgitation ratings are shown in Table 2. Table 3 shows the association of PAP elevation with survival and effect of engraftment in auto-HSCT patients. In auto-HSCT patients, 100-day mortality and 12-month mortality were not statistically significantly different between the groups with high PAP and normal PAP values (p>0.05). Five-year mortality rates were 48.6% (17 patients) and 10.9% (26 patients) in patients with a PAP of >25 mmHg and normal PAP values, respectively. This difference was statistically significant (p<0.05). Furthermore, 5-year survival was 38% (median: 33.6 months) in 35 patients with a PAP of >25 mmHg and 61% in the other group (Figure 1). This difference was statistically significant (p<0.05). When allo-HSCT transplant patients were evaluated, there was no statistically significant difference between the two groups in terms of overall survival rates (p>0.05) (Table 4).

## Discussion

There are many complications after HSCT. Cardiac complications rarely cause mortality and morbidity. Early cardiovascular complications may occur due to pre-transplant medical history, primary diagnosis, age, associated comorbidities, mobility regimen, and transplantation type [12]. Late cardiovascular complications may develop due to cardiotoxic chemotherapy exposure, radiotherapy, and age at the time of transplantation. In particular, post-transplant cardiovascular risk increases threefold in women, and in the auto-HSCT group, the risk of cardiac dysfunction increases fourfold [12]. Therefore, pre-transplant cardiac monitoring is routinely performed. A patient who is planned to undergo HSCT has adequate cardiac reserve for transplantation when the EF is above 35%-40% [13]. In our study, only 11 patients were found to have undergone transplantation with EF below 50%.

Murdych and Weisdorf [14] studied a total of 2821 patients who underwent HSCT. Cardiac complications were detected in 26 (0.9%) of these patients and 11 patients died due to HF. Decrease in EF can usually occur after treatment with cardiotoxic chemotherapeutic agents. In our study, we did not have death due to heart failure. Anthracycline-related HF risk is associated with a cumulative 500-550 mg/m^2^ dose in 4% and a cumulative 600 mg/m^2^ dose in 36%. For HSCT-treated patients, a cumulative dose of 250 mg is required for the threshold HF development limit [15]. We did not encounter anthracycline at doses exceeding the upper limits available in our study. Post-transplant beta-blockers can be used to reduce the cardiotoxic effects of anthracycline drugs [16].

The EF value increased after transplantation in 2 patients in each group. The association of this elevation with transplantation is regarded as insignificant for two reasons. The first is that no information was found on whether the patients received HF treatment, and, if so, how regular that treatment was. The second reason is that when the possibility of cardiac regeneration due to HSCT is emphasized, the probability of stem cells passing through the pulmonary artery and capillaries without any damage after transplantation is low [17].

A study by Dandoy et al. [8] revealed that symptoms of newly developed tachypnea, hypoxia, and respiratory failure occurred on the median 70th day. It was reported that the mortality rate in 40 cases presented in the literature was 55% and the cause of mortality was PH and its complications in 86% of these cases. A literature review reported that patients developed PH between 1 month and 51 years after transplantation (median: 12.6 years). In our study, no significant increase in mortality was found in the 100-day and 12-month follow-ups in both groups of allo-HSCT and auto-HSCT patients who had PAP values of >25 mmHg; however, overall 5-year survival was poor for auto-HSCT patients (p<0.001).

All pre-transplant patients should be evaluated with respiratory function tests. The FEV1 % value should be above 80% of the expected value, while carbon monoxide diffusion capacity (DLCO) should be greater than 50% of the expected value [18,19]. In our study, the FEV1 % value was a median of 83% (80%-95%) for patients with allo-HSCT and 89% (82%-97%) for patients with auto-HSCT.

The EBMT has established a risk classification for patients who have undergone allo-HSCT. The classification includes the age of the patient, the stage of disease, the type of donor, the time interval between diagnosis and transplantation, and a sex difference between donor and host [20]. In our study, we investigated the age and sex of patients and found that PAP values increase the mortality rate as an independent risk factor when age and sex are taken into the confidence interval in transplantation patients.

## Conclusion

The PAP value, which has not yet been investigated in acute and chronic cardiac complications, has emerged here as an important risk factor that can affect overall survival.

## Figures and Tables

**Table 1:**
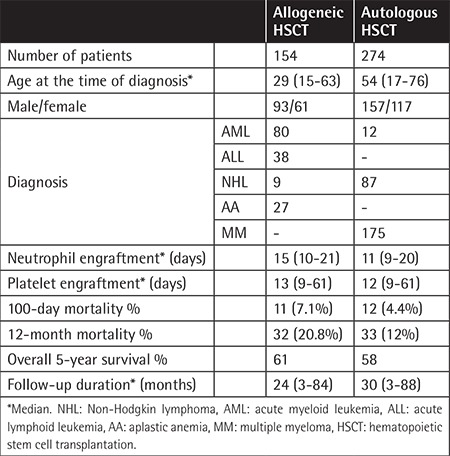
General characteristics of the patients undergoing hematopoietic stem cell transplantation.

**Table 2:**
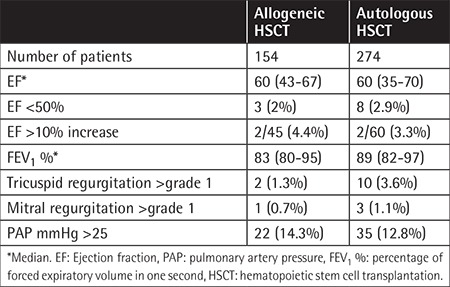
Pulmonary and cardiac characteristics of the patients undergoing hematopoietic stem cell transplantation.

**Table 3:**
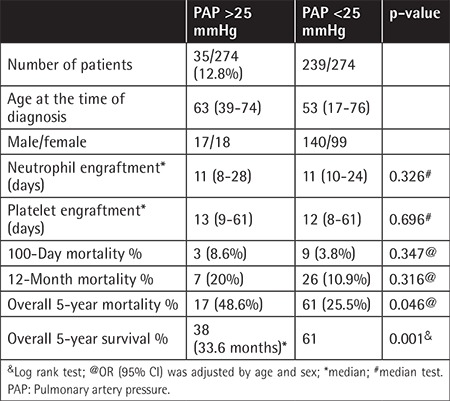
Association between pulmonary artery pressure and mortality and survival in patients who underwent autologous hematopoietic stem cell transplantation.

**Table 4:**
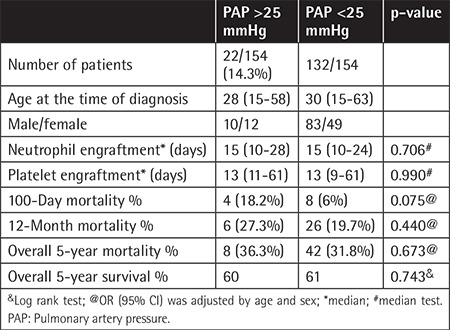
Association between pulmonary artery pressure and mortality and survival in patients who underwent allogeneic hematopoietic stem cell transplantation.

## References

[1] Ballen KK, Lazarus H (2016). Cord blood transplant for acute myeloid leukaemia. Br J Haematol.

[2] Murbraech K, Smeland KB, Holte H, Loge JH, Lund MB, Wethal T, Holte E, Rösner A, Dalen H, Kvaløy S, Falk RS, Aakhus S, Kiserud CE (2015). Heart failure and asymptomatic left ventricular systolic dysfunction in lymphoma survivors treated with autologous stem-cell transplantation: a national crosssectional study. J Clin Oncol.

[3] Lehmann S, Isberg B, Ljungman P, Paul C (2000). Cardiac systolic function before and after hematopoietic stem cell transplantation. Bone Marrow Transplant.

[4] McMurray JJ, Adamopoulos S, Anker SD, Auricchio A, Böhm M, Dickstein K, Falk V, Filippatos G, Fonseca C, Gomez-Sanchez MA, Jaarsma T, Køber L, Lip GY, Maggioni AP, Parkhomenko A, Pieske BM, Popescu BA, Rønnevik PK, Rutten FH, Schwitter J, Seferovic P, Stepinska J, Trindade PT, Voors AA, Zannad F, Zeiher A; Task Force for the Diagnosis and Treatment of Acute and Chronic Heart Failure 2012 of the European Society of Cardiology, Bax JJ, Baumgartner H, Ceconi C, Dean V, Deaton C, Fagard R, Funck- Brentano C, Hasdai D, Hoes A, Kirchhof P, Knuuti J, Kolh P, McDonagh T, Moulin C, Popescu BA, Reiner Z, Sechtem U, Sirnes PA, Tendera M, Torbicki A, Vahanian A, Windecker S, McDonagh T, Sechtem U, Bonet LA, Avraamides P, Ben Lamin HA, Brignole M, Coca A, Cowburn P, Dargie H, Elliott P, Flachskampf FA, Guida GF, Hardman S, Iung B, Merkely B, Mueller C, Nanas JN, Nielsen OW, Orn S, Parissis JT, Ponikowski P;, ESC Committee for Practice Guidelines (2012). ESC guidelines for the diagnosis and treatment of acute and chronic heart failure 2012: The Task Force for the Diagnosis and Treatment of Acute and Chronic Heart Failure 2012 of the European Society of Cardiology. Developed in collaboration with the Heart Failure Association (HFA) of the ESC. Eur J Heart Fail.

[5] Kim J, Shapiro L, Flynn A (2015). The clinical application of mesenchymal stem cells and cardiac stem cells as a therapy for cardiovascular disease. Pharmacol Ther.

[6] Pesto S, Begic Z, Prevljak S, Pecar E, Kukavica N, Begic E (2016). Pulmonary hypertension - new trends of diagnostic and therapy. Med Arch.

[7] Parasuraman S, Walker S, Loudon BL, Gollop ND, Wilson AM, Lowery C, Frenneaux MP (2016). Assessment of pulmonary artery pressure by echocardiography-A comprehensive review. Int J Cardiol Heart Vasc.

[8] Dandoy CE, Hirsch R, Chima R, Davies SM, Jodele S (2013). Pulmonary hypertension after hematopoietic stem cell transplantation. Biol Blood Marrow Transplant.

[9] Currie PJ, Seward JB, Chan KL, Fyfe DA, Hagler DJ, Mair DD, Reeder GS, Nishimura RA, Tajik AJ (1985). Continuous wave Doppler determination of right ventricular pressure: a simultaneous Doppler-catheterization study in 127 patients. J Am Coll Cardiol.

[10] Galiè N, Humbert M, Vachiery JL, Gibbs S, Lang I, Torbicki A, Simonneau G, Peacock A, Vonk Noordegraaf A, Beghetti M, Ghofrani A, Gomez Sanchez MA, Hansmann G, Klepetko W, Lancellotti P, Matucci M, McDonagh T, Pierard LA, Trindade PT, Zompatori M, Hoeper M (2015). 2015 ESC/ERS guidelines for the diagnosis and treatment of pulmonary hypertension: The Joint Task Force for the Diagnosis and Treatment of Pulmonary Hypertension of the European Society of Cardiology (ESC) and the European Respiratory Society (ERS): Endorsed by: Association for European Paediatric and Congenital Cardiology (AEPC), International Society for Heart and Lung Transplantation (ISHLT). Eur Respir J.

[11] Kinkaid HY, Huang XP, Li RK, Weisel RD (2010). What’s new in cardiac cell therapy? Allogeneic bone marrow stromal cells as “universal donor cells”. J Card Surg.

[12] Armenian SH, Sun CL, Shannon T, Mills G, Francisco L, Venkataraman K, Wong FL, Forman SJ, Bhatia S (2011). Incidence and predictors of congestive heart failure after autologous hematopoietic cell transplantation. Blood.

[13] Coghlan JG, Handler CE, Kottaridis PD (2007). Cardiac assessment of patients for haematopoietic stem cell transplantation. Best Pract Res Clin Haematol.

[14] Murdych T, Weisdorf DJ (2001). Serious cardiac complications during bone marrow transplantation at the University of Minnesota, 1977-1997. Bone Marrow Transplant.

[15] Armenian SH, Sun CL, Vase T, Ness KK, Blum E, Francisco L, Venkataraman K, Samoa R, Wong FL, Forman SJ, Bhatia S (2012). Cardiovascular risk factors in hematopoietic cell transplantation survivors: role in development of subsequent cardiovascular disease. Blood.

[16] Roziakova L, Mistrik M, Batorova A, Kruzliak P, Bojtarova E, Dubrava J, Gergel J, Mladosievicova B (2015). Can we predict clinical cardiotoxicity with cardiac biomarkers in patients after haematopoietic stem cell transplantation?. Cardiovasc Toxicol.

[17] Holinski S, Heinze G, Knebel F, Borges AC, Baumann G, Rudolph B, Konertz W (2012). Cardiac effects of experimental intravenous bone marrow cell transplantation after myocardial infarction. Ann Thorac Cardiovasc Surg.

[18] Folz RJ (2003). Allogeneic stem cell transplant, lung disease and airflow obstruction. Am J Respir Crit Care Med.

[19] Ho VT, Weller E, Lee SJ, Alyea EP, Antin JH, Soiffer RJ (2001). Prognostic factors for early severe pulmonary complications after hematopoietic stem cell transplantation. Biol Blood Marrow Transplant.

[20] Wang HT, Chang YJ, Xu LP, Liu DH, Wang Y, Liu KY, Huang XJ (2014). EBMT risk score can predict the outcome of leukaemia after unmanipulated haploidentical blood and marrow transplantation. Bone Marrow Transplant.

